# The burden of low back pain among undergraduate physiotherapy students at the University of Zimbabwe: a cross-sectional study

**DOI:** 10.1186/s13104-018-3796-5

**Published:** 2018-10-04

**Authors:** M. Chiwaridzo, K. J. Chamarime, J. M. Dambi

**Affiliations:** 1 Department of Rehabilitation, College of Health Sciences, University of Zimbabwe, Ground Floor, New Health Sciences Building, Avondale, Harare, Zimbabwe; 2 Mutare Provincial Hospital, P.O Box 30, Mutare, Harare, Zimbabwe

**Keywords:** Physiotherapy, Low back pain, Zimbabwe

## Abstract

**Objective:**

Globally, non-specific low back pain (NSLBP) is a common cause of morbidity in all people including physiotherapy students. However, no study has investigated the problem among undergraduate physiotherapy students in Zimbabwe. This study was conducted, therefore, to provide evidence of the prevalence, clinical characteristics and consequences of recurrent NSLBP among undergraduate physiotherapy students at the University of Zimbabwe.

**Results:**

The final sample had 90 participants, giving a study response rate of 97.8%. The median age of the participants was 22 years. The lifetime prevalence of NSLBP was 56.7% (n = 51) and the mean age of onset for NSLBP was 19.7 years (SD = 1.64 years). The 12-month prevalence of recurrent NSLBP was 38.9% (n = 35). Of the 35, 20 (57.1%) experienced at least three episodes in the last 12 months. Each episode lasted for 1–7 days in most participants (n = 31, 88.6%). The mean intensity of recurrent episodes was 3.37 (SD = 1.43) measured on Visual Analogue Scale. Only 7 (20%) experienced at least one functional limitation due to recurrent NSLBP. Additionally, only 2 (5.7%) sought medical treatment for the pain. However, 6 (17.1%) had to be absent from the university secondary to recurrent NSLBP.

## Introduction

Non-specific low back pain (NSLBP) is a major global burden indiscriminately affecting all people including physiotherapists [1–9]. Physiotherapists are at an increased risk of developing NSLBP due to the physical demands of the profession [7, 10–15]. This is despite physiotherapists having expert knowledge about injury prevention from training [16]. However, many physiotherapists report the first episodes of NSLBP during undergraduate training [11, 13, 14]. The possibility of university students being burdened with NSLBP before full-time employment is a cause of concern [3]. This is particularly so for physiotherapy students who are exposed to physical manual handling techniques during training and the risk factors for NSLB are likely to increase once the trainees qualify and assume full-time jobs which are more demanding [17].

Different studies have reported varying prevalence rates for NSLBP among physiotherapy students with lifetime prevalence figures ranging between 36 and 69% [7, 10, 12, 17]. Also, Nyland and Grimmer [12] reported a 12-month prevalence of 63% in Australia. However, there is dearth of literature on the prevalence of NSLBP among undergraduate physiotherapy students from low-resourced settings like Zimbabwe. This is a significant shortcoming against the background of high prevalence of occupational-related NSLBP reported among Zimbabwean physiotherapists [15, 18]. Unabated, NSLBP has been linked to reduced health-related quality of life, school absenteeism, increased health-seeking behaviour and use of pain medication [19, 20]. Therefore, research efforts investigating the prevalence, clinical characteristics and consequences of recurrent NSLBP among undergraduate physiotherapy students are warranted to understand the magnitude of the condition, its impact and possibly inform primary prevention programmes. This study sought to (i) to determine the lifetime and 12-month prevalence of recurrent NSLBP among undergraduate physiotherapy students, (ii) to describe the clinical characteristics of recurrent NSLBP and lastly, (iii) to determine the consequences of recurrent NSLBP.

## Main text

### Study design, research setting and participants

A cross-sectional descriptive study was conducted at UZ targeting all undergraduate physiotherapy students. UZ is the only university offering undergraduate training in physiotherapy in Zimbabwe. The following parameters were used to estimate sample size using EPI info StatCalc: (i) total population of physiotherapy students at UZ (N = 92) (ii) expected 12-month prevalence for NSLBP of 32.5% [10], (iii) precision effect of 3%, (iv) design effect of 1. The minimum calculated sample size at 95% confidence interval was 84 students. All undergraduate physiotherapy students from first to final year were invited to participate. Physiotherapy is offered as a four-year long degree programme [21]. Exclusion from the study was based on students having a physical disability such as leg length discrepancy which is a known risk factor for low back pain [22].

### Survey instrument

The study questionnaire was adopted from previous studies [23, 24]. Briefly, the first section elicited demographic and university-related information. Section B had questions on lifetime prevalence, age at onset for the first episode of NSLBP, and recurrent NSLBP. Subsequent questions elicited information on the clinical characteristics of recurrent NSLBP guided by the definition provided by Stanton et al. [25]. Additionally, the questionnaire asked about the consequences of recurrent NSLBP. Nine questions derived from the Hanover Low Back Pain Disability Questionnaire enquired about functional activities limited [26–28]. Prior to use, the questionnaire was assessed for logical validity by five (5) experts as described elsewhere [26]. The questionnaire yielded excellent Scale/Average Content Validity Index (S-CVI/Ave = 0.97) [29, 30]. The questionnaire was evaluated for reproducibility among 16 occupational therapy students and showed kappa coefficients between 0.4 and 1, indicating fair to perfect agreement assessments [31].

### Procedure

This study adhered to ethical principles under the Declaration of Helsinki [32]. Ethical clearance was obtained from the Joint Research Ethics Committee for the UZCHS and Parirenyatwa Group of Health Sciences (JREC REF: 281/6) and Medical Research Council of Zimbabwe (MRCZ/B/1213). Data were collected in February 2017. Consenting participants firstly signed informed consent before completing the self-administered questionnaire.

### Statistical analyses

The Shapiro–Wilk test assessed normality of continuous variables. Participants’ demographic and university-related information were analysed using descriptive statistics. Mann–Whitney U test assessed for differences in participants ages by gender. The intensity of recurrent NSLBP was evaluated using Visual Analogue Scale (VAS). The independent *t* test assessed for significant differences in the mean pain intensity between the sexes. Chi square test evaluated for an association between NSLBP and gender, age of the participants, place of residence and level of study (p ≤ 0.05). Data were analysed using Statistica version 13.2.

## Results

Of the 92 students, 90 (97.8%) responded and had a median age of 22.0 years (Interquartile range, IQR = 21–22 years). The final sample had 61 (67.8%) females. Males were significantly older compared to females (U = 581, p = 0.009). Most participants stayed in UZ halls of residences (n = 52, 57.8%). There were an almost equal number of third year students as there were fourth year students (Table 1). The lifetime prevalence of NSLBP was 56.7% (n = 51). The mean age of onset of NSLBP was 19.7 (SD = 1.64) years with no significant differences between sexes [t (49) = 1.63, p = 0.11]. Lifetime prevalence was associated with residing in UZ residences [X^2^(1) = 7.92, p = 0.005] but not with gender [X^2^(1) = 0.43, p = 0.51], and year of study [X^2^(3) = 2.06, p = 0.56].

**Table 1:** Recurrent non-specific low back pain and socio-demographic associated factors (N = 90)

Characteristics	Frequency, n	LBP n (%)	No LBP n (%)	Chi square	P value
Gender					
Male	29	13 (44.8)	16 (55.2)	X^2^(1) = 0.63	0.43
Female	61	22 (36.1)	39 (63.9)		
Age (years)					
19	3	1 (33.3)	2 (66.7)	${\text{X}}_{{({\text{linear}}\;{\text{trend}})}}^{2}$ = 8.38	0.21
20	17	3 (17.6)	14 (82.4)		
21	17	8 (47.1)	9 (52.9)		
22	33	13 (39.4)	20 (60.6)		
23	11	5 (45.5)	6 (54.5)		
24	8	5 (62.5)	3 (37.5)		
25	1	0 (0)	1 (100)		
Year of study					
First year	21	5 (23.8)	16 (76.2)	X^2^(3) = 2.03	0.57
Second year	14	6 (42.9)	8 (57.1)		
Third year	28	11 (39.3)	17 (60.7)		
Fourth year	27	13 (48.1)	14 (51.9)		
Place of residence					
UZ residence	52	26 (50.0)	26 (50.0)	X^2^(1) = 6.40	0.01
Outside UZ residence	38	9 (23.7)	29 (76.3)		

*UZ* University of Zimbabwe

The 12-month prevalence of recurrent NSLBP was 38.9% (n = 35). Of the 35, 20 (57.1%) experienced at least three episodes of recurrent NSLBP in the last 12 months. Each episode lasted 1–7 days in most participants (n = 31, 88.6%). The mean intensity of recurrent NSLBP episodes was 3.37 (SD = 1.43) on the Visual Analogue Scale (VAS) with no significant difference between sexes [t (33) = 0.04, p = 0.96]. Only 7 (20%) experienced at least one functional limitation in activities due to recurrent NSLBP. Additionally, 2 (5.7%) students sought medical treatment for the pain and 6 (17.1%) reported absenteeism from the university at one point in time due to recurrent NSLBP. Recurrent NSLBP was neither associated with gender nor year of study but was associated with lifetime NSLBP [X^2^(1) = 43.8, p < 0.01] and place of residence [X^2^(1) = 6.40, p = 0.01] (Table 1). Students residing at UZ reported more recurrent NSLBP. Although the 12-month prevalence increased with age, this was not statistically significant [${\text{X}}_{{({\text{linear}}\;{\text{trend}})}}^{2}$ = 8.38, p = 0.21].

## Discussion

Although NSLBP is a common occurrence among undergraduate physiotherapy students worldwide [7, 10, 12, 17], nothing is known about the prevalence, clinical characteristics and consequences of the condition in the Zimbabwean setting. Similar studies in Zimbabwe investigated the burden of work-related musculoskeletal disorders (WMSDs) among qualified physiotherapists [15, 18]. Results from these studies cited NSLBP as the most common occupational injury experienced especially by young qualified physiotherapists. This concurs with the present study as more than half (56.7%) of the students reported having experienced at least one episode of NSLBP in their lifetime. This finding indicates that NSLBP is a common occurrence among Zimbabwean physiotherapy students. Studies elsewhere have reported comparable and contrasting findings [7, 11, 12, 17, 33]. Consistently, Horrell et al. [17] reported lifetime prevalence of 62.3% among physiotherapy students at one university in England. NSLBP was less common among South African undergraduate physiotherapy students with lifetime prevalence of 36% [7]. Additionally, physiotherapy students in Brazil were highly affected with 82.3% lifetime prevalence [11]. It appears that the variations in the lifetime prevalence figures between studies mainly reflect population and methodological differences [7].

In our study, the mean age of onset for an episode of NSLBP was 19.7 years implying that most students experienced their first episode of NSLBP soon after enrolling for physiotherapy. This finding suggests that studying physiotherapy is a potential risk factor for the development of NSLBP. This warrants longitudinal investigation into the factors associated with the occurrence of NSLBP among Zimbabwean undergraduate physiotherapy students. Elsewhere, the development of NSLBP among physiotherapy students has been linked with cumulative hours of practical exposure and demographic factors such as gender [7, 11]. Practical work in physiotherapy often involves use of physical manual techniques with performance of repetitive movements, prolonged standing, lifting, transferring and adoption of awkward postures [10]. Constant exposure coupled with inherent biological or gender differences probably account for the reported link between practical exposure, gender and NSLBP. However, in the present study, lifetime prevalence was not associated with gender (p = 0.51) and year of study (p = 0.56). This possibly highlights the indiscriminate nature of NSLBP equally affecting both sexes and all students at different levels of study and these findings are consistent with other studies [10].

A small subset of undergraduate physiotherapy students (38.9%) reported recurrent NSLBP in the last 12 months. This finding adds support to the fact that NSLBP is recurrent [25]. Recurrence rate of NSLBP among physiotherapy students was slightly higher compared to rates reported in other occupational groups in Zimbabwe. For instance, NSLBP was found to be recurrent in 30.7% of Zimbabwean high school-children [34]. Although these studies are incomparable because of different target population, the results of the present study potentially suggest greater exposure to occupational risk factors for NSLBP in physiotherapy students. Recurrent NSLBP may indeed be a problem among Zimbabwe undergraduate physiotherapy students considering also the reported 12-month prevalence of occupational NSLBP reported in qualified physiotherapists in the country. For example, Useh et al. [15] reported a comparable prevalence of 52.1% for LBP. There are few studies documenting the 12-month prevalence of NSLBP among undergraduate physiotherapy students and the prevalence figures have been shown to vary [10, 12, 13, 33]. Consistent with present study findings, Vincent-Onabajo et al. [10] reported a 12-month cross-sectional prevalence of 32.5% among Nigerian physiotherapy students. Other studies from Brazil and Pakistan reported relatively higher prevalence values above 70% [11, 33].

The present study found that more than half of the students had at least three episodes of NSLBP in the last 12 months. Although the episodes were frequent, most were mild in intensity and lasted only for a short duration. These findings are interesting and suggest a favourable natural trajectory of NSLBP in young adults. Additionally, these finding highlight the benign nature of NSLBP experienced by physiotherapy students and similar outcomes have been reported in school-children [6]. However, the fact that the episodes were frequent shows possible constant exposure of the students to occupational risk factors associated with continued development of NSLBP. All these results possibly explain reduced school absenteeism rates among students who had recurrent NSLBP. Additionally, it is possible that the benign nature of the recurrent NSLBP account for reduced seeking medical professional help among the students (n = 2, 5.7%).

In conclusion, this present study showed that NSLBP is a common occurrence among undergraduate physiotherapy students at the UZ and in a small subset of students the condition is recurrent. Fortunately, the condition seems benign in nature with limited consequences on students.

## Limitations

This study had the following limitations

i. The study was conducted as a cross-sectional study relying on self-reported data. Therefore, causality cannot be established between the factors identified to be associated with NSLBP.
ii. Recall bias and forward telescoping may have led to under-or-over-estimation of the prevalence figures [35].
iii. Data collection was conducted by the second author (KJC) who was a final undergraduate physiotherapy student at the time. The questionnaires were self-administered and questionnaires were collected immediately upon completion. Possibly, this could have influenced the results.

## Abbreviations

IQR: interquartile range
LBP: low back pain
NSLBP: non-specific low back pain
SD: standard deviation
WMSDs: work-related musculoskeletal disorders
VAS: Visual Analogue Scale
UZCHS: University of Zimbabwe College of Health Sciences
